# A second iron injection administered to piglets during lactation improves hemoglobin concentration, growth performance, and carcass characteristics at slaughter

**DOI:** 10.1093/jas/skad270

**Published:** 2023-08-10

**Authors:** Tyler B Chevalier, Wesley Lyons, Duncan B Paczosa, Gregg K Rentfrow, Merlin D Lindemann

**Affiliations:** Department of Animal and Food Sciences, University of Kentucky, Lexington, KY 40546, USA; Pharmacosmos Inc., Watchung, NJ, USA; Department of Animal and Food Sciences, University of Kentucky, Lexington, KY 40546, USA; Department of Animal and Food Sciences, University of Kentucky, Lexington, KY 40546, USA; Department of Animal and Food Sciences, University of Kentucky, Lexington, KY 40546, USA

**Keywords:** carcass, hemoglobin, injection, iron dextran, piglet, weaning

## Abstract

An experiment was conducted to evaluate the effects of a second injection of iron dextran administered on days 6 to 8 of age. A total of 144 crossbred pigs (equal barrows and gilts; initial age 6 to 8 d; initial body weight [**BW**] = 2.86 ± 0.01 kg) were assigned to either the control (CON) or an added-injection treatment (+Fe). Pigs were paired by sex and BW within a litter and randomly assigned to the iron treatment within each pair. All pigs had received an initial intramuscular (**IM**) injection of iron dextran (200 mg Fe) <24 h after birth. Pigs assigned to the +Fe treatment received a second IM injection of iron dextran (200 mg Fe) on days 6 to 8. All pigs were weaned at 22 to 25 d, housed 6 pigs/pen, and received a common corn–soybean meal diet. BW and feed disappearance were recorded every 2 wk. Hemoglobin (**Hb**) concentrations were measured at birth, initiation of experiment (days 6 to 8), weaning, and the end of the nursery and end of the study. At the end of the study, 1 pig/pen (*n* = 12 pigs/treatment), closest to the pen mean was selected and slaughtered for carcass characteristic measures. The individual pig served as the experimental unit for BW, Hb, average daily gain (**ADG**), and carcass characteristic data whereas the pen served as the experimental unit for average daily feed intake, and gain/feed ratio data. The +Fe pigs had a greater Hb at weaning (13.1 vs. 10.7 g/dL, respectively; *P <* 0.01) and end of the nursery (12.1 vs. 11.7 g/dL, respectively; *P* = 0.01) compared to CON pigs. During the finisher period, +Fe pigs had a greater ADG (0.94 vs. 0.91 kg, respectively; *P* = 0.05) compared to CON pigs. Overall, pigs receiving the second iron injection had an ~4% increase in ADG (*P* = 0.04) from weaning to the end of study. The cumulative improvement in ADG from weaning to the end of study observed for +Fe group resulted in +Fe pigs having a heavier BW at the end of the study (~3 kg; *P* = 0.04). Following slaughter, +Fe pigs had ~7.2% heavier trimmed loin (*P* = 0.04) compared to the CON pigs. In conclusion, administering a second iron injection resulted in greater Hb at weaning and the end of the nursery as well as improved growth performance from weaning to the end of study weight and increased carcass weight at slaughter.

## Teaser

Administering a second iron injection to pigs before weaning resulted in greater Hb levels at weaning and the end of the nursery period.

Average daily gain from weaning to slaughter was greater for pigs receiving a second iron injection compared to only the initial injection at litter processing.

## Introduction

It is routine practice in commercial swine production to provide newborn piglets with 100 to 200 mg of iron via an intramuscular (**IM**) injection shortly after birth. The purpose of the practice is to prevent iron deficiency anemia, because pigs are born with limited stores of iron and only receive ~1 mg of iron per day through the sow milk (Venn et al., 1947). Previous literature has suggested that the iron requirement of piglets during lactation is mainly dependent on the growth rate of the piglet (Venn et al., 1947; Kamphues et al., 1992; Egeli and Framstad, 1998; Van Gorp et al., 2012). Other work has demonstrated that the industry standard iron injection (100–200 mg Fe) administered to piglets shortly after birth is not sufficient to maintain the iron status of the pig throughout the lactation period(Bhattarai and Nielsen, 2015a; Perri et al., 2016). Unsurprisingly, faster-growing pigs in a litter are most susceptible to becoming iron deficient at weaning creating potential postweaning problems (Jolliff and Mahan, 2011).

Recently, Chevalier et al. (2021) demonstrated that pigs administered 200 mg Fe at birth had peak hemoglobin (**Hb**) concentration on day 17 that subsequently declined to weaning on day 22. Furthermore, pigs that only received 100 mg Fe at birth had peak Hb on day 11 with a decline in Hb thereafter. These results agree with previous literature which suggests a possibility of pigs having suboptimal hemoglobin concentrations (generally <11 g/dL as defined by Thorn, 2010; Bhattarai and Nielsen, 2015b; Perri et al., 2016) at weaning. Interestingly, it has been demonstrated that pigs with optimal hemoglobin concentrations at weaning have greater growth performance in the subsequent postweaning periods (Bhattarai and Nielsen, 2015b; Fredericks et al., 2018). Furthermore, Olsen (2019) demonstrated that an additional iron injection given to piglets 4 to 6 d after the initial iron injection resulted in a much larger percentage (89% vs. 20%) of pigs having optimal Hb concentration (≥11 g/dL) at weaning and a 454 g increase in body weight (**BW**) 42 d postweaning. Thus, there is a continued need to reevaluate iron supplementation practices as there may be a potential benefit to administration of a second iron injection before weaning. Thus, the current experiment evaluated a second iron injection administered before weaning on hemoglobin concentration, growth performance, and carcass responses from lactation through the finishing period.

## Materials and Methods

This experiment was carried out in an environmentally controlled room at the University of Kentucky Swine Research Center. The experiment was conducted under protocols approved by the Institutional Animal Care and Use Committee of the University of Kentucky.

### Animals and experimental design

A subset of 144 crossbred pigs (Yorkshire × Landrace × Large White; 72 barrows and 72 gilts; initial BW = 2.86 ± 0.01 kg) from a total 21 of litters were assigned to either a control (CON) or added-injection treatment (+Fe) on days 6 to 8 of age. At enrollment (days 6 to 8), pairs were created by pairing two littermates (siblings) with the same sex that had a BW difference of <0.23 kg. Within each pair, one pig was assigned to the CON and the other to +Fe group. All pigs were processed (clipping needle teeth, docking tails, and ear-tagged) <24 h following birth and received an initial 200 mg intramuscular (**IM**) iron injection (Uniferon, Pharmacosmos Inc., Watchung, NJ). The pigs assigned to the +Fe group received a second 200 mg Fe IM injection on days 6 to 8. All male pigs were castrated prior to enrollment on days 6 to 8.

All pigs were weaned on days 22 to 25 to a nursery facility and housed 6 pigs/pen (1.22 × 2.44 m^2^). Pigs were placed in pens consisting of the same treatment and similar BW; thus, the six heaviest pairs of pigs created two pens, one for the control treatment and one for the second injection treatments. A total of 12 pens resulted, six of control pigs and six of +Fe pigs. In the nursery and grow-finish periods, both CON and +Fe pigs received a common corn–soybean meal diet formulated to meet or exceed the NRC (1998) requirement estimates relative to BW until the end of study (Table 1). The common diet fed for the duration of the experiment was formulated to supply an added 100 mg/kg Fe as ferrous sulfate. Pigs were transitioned from a nursery to a finisher facility at the end of week 5 while maintaining the same pen structure but provided with more space (1.80 × 3.04 m^2^). All pigs had ad libitum access to feed and water throughout the entire experiment.

**Table 1.** Diet formulation and composition of diets fed from weaning to the end of study (as-fed basis)

Item	7 to 25 kg	25 to 50 kg	50 to 75 kg	75 to 135 kg
Ingredient, %				
Corn	56.47	70.33	76.43	81.63
Soybean meal, 48% CP	32.50	26.00	20.00	15.00
Grease, choice white	2.50	1.00	1.00	1.00
Whey dried	5.00	—	—	—
l-Lysine·HCl	0.24	—	—	—
dl-Methionine	0.13	0.03	—	—
l-Threonine	0.14	0.03	—	—
Dicalcium phosphate	1.30	0.90	1.00	0.90
Limestone	1.00	1.00	0.85	0.75
Salt	0.50	0.50	0.50	0.50
Trace mineral premix^1^	0.10	0.10	0.10	0.10
Vitamin premix^2^	0.10	0.10	0.10	0.10
Santoquin^3^	0.02	0.02	0.02	0.02
Total	100.00	100.00	100.00	100.00
Calculated nutrient level, %				
ME, Kcal/kg	3,369	3,326	3,333	3,345
Crude protein	21.18	18.24	15.84	13.89
SID lysine^4^	1.19	0.82	0.67	0.55
Ca	0.80	0.66	0.61	0.53
Total P	0.65	0.53	0.53	0.49
STTD P^5^	0.44	0.32	0.32	0.29

^1^Provided per kilogram of total diets: 50 mg Mn as manganous sulfate, 100 mg of Fe as ferrous sulfate, 125 mg of Zn as zinc sulfate, 18 mg of Cu as copper sulfate, 0.35 mg of I as calcium iodate, and 0.30 mg of Se as sodium selenite.

^2^Provided per kilogram of total diet: 11,025 IU of vitamin A; 1,103 IU of vitamin D3; 77 IU of vitamin E; 2.2 IU of vitamin K; 0.03 mg of vitamin B12; 0.36 mg of biotin; 4.96 mg of folic acid; 30.32 mg of niacin; 27.56 mg of pantothenic acid; 4.96 mg vitamin B6 and 1.65 mg thiamin; and 8.27 mg of riboflavin.

^3^Santoquin (Monsanto, St. Louis, MO, USA) supplied 130 mg/kg ethoxyquin to the final diet.

^4^SID, standardized ileal digestible.

^5^STTD, standardized total tract digestible.

### Measurements and sample collection

#### Hemoglobin and growth performance

Hemoglobin concentration (Hb) was measured at birth, enrollment, weaning, and the end of the nursery and the end of study by using a HemoCue Hb 201+ analyzer (HemoCue America, Brea, California). The HemoCue Hb 201+ was previously validated to assess Hb in arterial blood of pig (Kutter et al., 2012). Blood samples were taken from the ear veins of the pigs before the initial and second iron injections were administered and loaded into disposable microcuvettes via capillary action. The microcuvette was placed in the HemoCue Hb 201+ and the resulting Hb concentration displayed and recorded within 60 s. Body weight and feed disappearance were recorded every 2 wk to determine average daily gain (**ADG**), average daily feed intake (**ADFI**), and gain-to-feed ratio (**G/F**).

#### Harvest and carcass characteristics

One pig/pen that was closest to the pen mean was slaughtered at ~21 wk of age under USDA inspection. After being transferred to the University of Kentucky Meat Science Laboratory, the pigs were slaughtered after a rest of at least 30 min. The slaughter process included electrical stunning, exsanguination, dehairing, evisceration, and carcass washing. During the process of slaughter, organs including the liver and spleen were obtained and weighed. Then, 45-min pH of longissimus muscle (LM) at the 10th rib was measured in the meat cooler with an Accumet 50 pH meter (Fisher Scientific, Fairlawn, NJ, USA).

The hot (**HCW**) and cold carcass weight (**CCW**), carcass length, backfat depth at four locations (1st rib, last rib, 10th rib, and last lumbar), *longissimus dorsi* muscle area (**LMA**), and 24-h pH were measured according to the methods described by McClelland et al. (2012). Briefly, HCW was recorded immediately after harvest to calculate dressing percentage [(HCW/BW) × 100]. Following a 24-h chill (4 °C), CCW, backfat depth at the 10th rib, 1st rib, last rib, and last lumbar were measured. Shrink loss (%) was calculated using HCW and CCW, with the equation {[1 − (CCW/HCW)] × 100}. Carcass length was measured from the anterior edge of the symphysis pubic to the recess of the first rib.

The LMA and 24-h pH were measured from the left side of each carcass according to methods described by National Pork Producers Council (NPPC, 2000). The Accumet 50 pH meter was used for the 24-h pH. After a 24-h cooler chill, primal cuts were separated and weighed individually according to Institutional Meat Purchasing Specifications (IMPS), including Boston butt (IMPS #406), shoulder picnic (IMPS #405), loin (IMPS #410), belly (IMPS #408; squared at each end), and spareribs (North American Meat Processors Association, 2010).

#### Meat quality

As soon as primal cut measurements were finished, slices of LM (~1.2 cm in thickness and around 100 g) were obtained posterior to the 10th rib location. Drip loss was determined by suspending the sample, covered with a black plastic bag, from a hook in darkness, and stored at 4 °C for 48 h. The samples were weighed before and after the process, drip loss percentage was calculated by the 48 h weight loss divided by the initial weight × 100. About 10-cm chops of loin samples posterior to the 10th and 11th rib interface were obtained and weighed prior to being vacuum packaged, boxed, and stored under refrigeration (4 °C) for 30 d to simulate the period of time between the packing plant and the retail grocery store. Loin samples were reweighed on days 7, 14, and 30 to determine purge loss at each stage to help determine when the majority of the weight is lost during storage. Purge loss percentage was calculated by the weight loss divided by initial weight × 100. Each time the samples were weighed, loin samples were taken out of the vacuum package, surface water was removed with a paper towel, the weight was recorded, and then the loin samples were vacuum packaged again.

Another 2.54-cm loin chop sample was cut from the LM between the 7th and 8th ribs. These loin samples were placed on foam trays and overwrapped in polyvinyl chloride (PVC) film. Subjective color, firmness, and marbling scores (NPPC, 2000) were evaluated by a trained experienced expert. The NPPC color scale (1 to 5) was used for color evaluation: 1 = pale pinkish to white; 5 = dark purplish red. Similarly, the NPPC marbling scale (1 to 5, percentage fat in the LM) and NPPC firmness scale (1 to 5, 1 = very soft; 5 = very firm) were used. Afterward, trays were then placed under cool white fluorescent lighting (1,200 lux) at 4 °C to mimic retail conditions. Objective color measurements were made using a Hunter Lab Miniscan XE Plus colorimeter (Hunter Lab Associates, Reston, VA, USA) with the L*, a*, and b* scale at D65 light source, 2.54 cm diameter aperture, and 10° standard observer. The instrument was standardized before the analysis with black and white tilers that had been overwrapped with PVC film to adjust for the PVC cover upon the meat. Spectral reflectance was determined every 10 nm over the 400 to 700 nm range. Observations were made as soon as the fabrication of the carcass was finished, and the meat color measurement was treated as the day 0 meat color.

### Statistical analysis

Prior to analysis, all data were initially evaluated for any potential statistical outliers by identifying any values that were three standard deviations from the mean of the response measure of interest. After reviewing experimental notes, a final decision whether to exclude the value was made. However, there were no statistical outliers determined for this experiment.

Data analyses were performed in SAS 9.4 (SAS Inst. Inc., Cary, NC, USA) by least-squares analysis of variance using the generalized linear model as a randomized complete block design. The individual pig served as the ­experimental unit for BW, ADG, carcass, and Hb data whereas the pen served as the experimental unit for pen ADG, ADFI, and gain/feed ratio. The statistical model included sex, treatment, pair, sex × treatment, and allotment pair for BW, ADG, Hb, and carcass characteristics. For ADFI, and gain/feed ratio the statistical model only included effects for sex, treatment, and sex × treatment. Statistically significant differences were established at *P* ≤ 0.05, tendencies were established at *P* ≤ 0.10 for main effects. When present (*P* ≤ 0.05), the effect of sex for each measurement is in the footnote of the table.

## Results and Discussion

There were expected sex effects observed for ADG, ADFI, and some carcass measures but no sex × treatment interactions for any response measures.

### Hemoglobin and growth performance

The mean Hb value for CON pigs at weaning was 10.7 g/dL (Table 2), which is below the often-recognized critical value of 11 g/dL (Thorn, 2010; Bhattarai and Nielsen, 2015b; Perri et al., 2016) and demonstrated that greater than 50% of the CON pigs would be considered either sub-clinically iron deficient or clinically anemic at weaning. Moreover, administering a second Fe injection on days 6 to 8 resulted in +Fe pigs (13.1 g/dL) exhibiting Hb levels much greater than the previously defined critical level, in addition to having a 22% higher Hb compared to their counterparts (13.1 vs. 10.7 g/dL, *P* < 0.01). Although less pronounced, the advantage observed in Hb at weaning remained through the end of the nursery period (12.1 vs. 11.7 g/dL, *P* = 0.01). The current findings are supported by previous work of Chevalier et al. (2021) where pigs administered a 200-mg Fe dextran injection within 24 h after birth had a peak Hb response on day 17 and had dropped below the Hb critical level (10.9 g/dL) by weaning (day 22) compared to pigs administered 300 mg Fe dextran having a Hb level above the critical limit (12.1 g/dL) at weaning. Interestingly, earlier work by Chevalier and Lindemann (2019) demonstrated that a second Fe injection provided 4 d before weaning resulted in greater Hb concentrations through the first 14 d following weaning but by days 27 to 30, there were no differences in Hb concentrations compared to pigs administered only a single Fe injection at processing. However, Chevalier and ­Lindemann (2019) only supplied 150 mg Fe at birth and then an additional 150 mg Fe 4 d prior to weaning for the +Fe pigs, whereas the current study supplied 200 mg Fe at birth and an additional 200 mg Fe 6 to 8 d after birth. Thus, one may speculate that the greater amount of total Fe administered to the pig before weaning (400 mg Fe in the current study vs. 300 mg Fe in the previous study) resulted in an extended duration of Hb being elevated over the controls. Agreeing with the current study, a study utilizing another form of iron (i.e., gleptoferron) that injected piglets with an initial 200 mg Fe IM injection on day 3 followed by an additional 100 mg Fe IM injection on day 11 resulted in pigs having a greater Hb level at weaning (day 21) and day 35 compared to piglets only receiving the initial 200 mg Fe injection (Williams et al., 2020). Despite the differences in dosage, dosage timing, and sampling timepoints between the study herein and Williams et al. (2020), both suggest that an additional iron injection before weaning leads to greater Hb levels at weaning and in the nursery period. By the end of the study there was no difference in Hb between the +Fe and CON groups (*P* = 0.66).

**Table 2.** Effects of a second iron injection administered on days 6 to 8 on hemoglobin (Hb) concentration^1^

Item	Treatment		SEM	*P*-value
	CON	+Fe		
Hb, g/dL				
Birth (day 0)^S^	9.9	9.9	0.21	0.93
Enrollment (days 6 to 8)	9.1	8.9	0.12	0.13
Weaning (days 22 to 24)	10.7	13.1	0.15	<0.01
Nursery^2^	11.7	12.1	0.13	0.01
End of study	11.9	12.0	0.14	0.66

^1^Data represents 144 pigs or 72 pigs/treatment. All pigs received an initial iron injection (200 mg Fe) at birth; the +Fe group received a second iron injection (200 mg Fe) at enrollment.

^2^Nursery period was at the end of week 5 postweaning.

^S^Significant sex effect present where males had a greater Hb at birth compared to females, *P* ≤ 0.05.

Individual pig BW and ADG are reported in Table 3. During the finisher period, +Fe pigs had a greater ADG (*P* = 0.05) compared to CON pigs. In general, pigs receiving the second iron injection had an ~4% increase in ADG (*P* = 0.04) from weaning to the end of study. The cumulative improvement in ADG from weaning to end of study resulted in +Fe pigs having a heavier BW at the end of the study (115.77 vs. 112.79 kg; *P* = 0.04).

**Table 3.** Effects of a second iron injection administered on days 6 to 8 on individual pig bodyweight and average daily gain^1^

Item	Treatment		SEM	*P*-value
	CON	+Fe		
Bodyweight, kg				
Birth (day 0)	1.53	1.55	0.01	0.53
Enrollment (days 6 to 8)	2.87	2.85	0.01	0.44
Weaning (days 22 to 25)	7.44	7.40	0.06	0.67
Week 2	10.51	10.85	0.11	0.03
Week 4	19.08	19.96	0.22	0.01
Week 5	27.63	28.15	0.27	0.17
Week 7	40.10	40.77	0.35	0.18
Week 9	53.69	54.72	0.49	0.13
Week 11	67.28	68.49	0.60	0.15
Week 13	81.03	82.70	0.76	0.11
Week 15	91.77	93.63	0.83	0.11
Week 17	103.89	106.88	0.97	0.03
End of study	112.79	115.77	1.03	0.04
Average daily gain, kg				
Preweaning^2^	0.29	0.29	0.004	0.73
Nursery^3^	0.55	0.56	0.01	0.10
Finisher^4^	0.91	0.94	0.01	0.05
Wean to end of study	0.81	0.83	0.01	0.04
Enrollment to end of study	0.75	0.78	0.01	0.04

^1^Data represents 144 pigs or 72 pigs/treatment. All pigs received an initial iron injection (200 mg Fe) at birth; the +Fe group received a second iron injection (200 mg Fe) at enrollment.

^2^Preweaning period included enrollment to weaning.

^3^Nursery period included weaning to week 5 postweaning.

^4^Finisher period included week 5 postweaning to the end of study.

It has been hypothesized that optimizing the hemoglobin concentration and the overall iron status of pigs can promote maximum immunity thereby increasing the health status of pigs (Perrin et al., 2016). Optimizing the health status of piglets before weaning can be a major contributor to subsequent growth performance in the nursery as this transition period can be very stressful for young pigs. Work conducted by Fredericks et al. (2018) revealed that pigs with optimal hemoglobin status (>11 g/dL) at weaning had a greater BW at 8 wk postweaning in contrast to pigs with lower hemoglobin concentrations (<11 g/dL). Earlier work by Kamphues et al. (1992) demonstrated that an additional Fe injection administered 1 wk before weaning increased daily BW gain through 3 wk postweaning (380 vs. 362 g/d) compared to pigs administered a single Fe injection. These reports are supportive of the results herein as the +Fe pigs had above optimal Hb concentrations at weaning and subsequently resulted in the improved ADG ultimately resulting in a heavier final BW. Conversely, other work by Williams et al. (2021) found no difference in final BW and ADG through market weight for pigs that were administered an additional iron injection on day 12. However, the differences between studies may be a result of the form of Fe supplemented or timing. Unlike the study herein that supplied 200 mg Fe via iron dextran, Williams et al. (2021) supplied 200 mg Fe via gleptoferron. In addition, the study herein supplied the additional iron injection on days 6 to 8 compared to day 12 in the previous report.

More recent work conducted by Chevalier and Lindemann (2019) demonstrated that a second iron injection provided 4 d before weaning resulted in a BW difference of ~1 kg by the end of 4 wk postweaning. In the current study, there was only an ~0.5 kg BW difference between treatment groups which was also recorded 1 wk later than the previously cited study. Moreover, the pigs in the current experiment were weaned at an older age and heavier BW; perhaps both of which contribute to a greater requirement in Fe, thus increasing the potential for a response associated with the increase in injectable Fe dosage between the current and previous study. However, the difference in the magnitude of response (+0.5 kg) between Chevalier and Lindemann (2019), and the experiment herein may raise questions regarding the optimal time for administration of an additional iron injection prior to weaning. Thus, supplying the additional Fe injection at an older age or closer to the expected decline in Hb before weaning (demonstrated by Chevalier et al., 2021) may provide a greater potential for improved ADG and BW.

Regarding pen growth performance (Table 4), there were sex effects (*P* < 0.05) observed for both ADG and ADFI during the finisher and wean to the end of study period in which the barrows had greater ADG and ADFI compared to the gilts. Although not statistically significant, on a pen basis +Fe pigs had a 3 and 1% greater ADG and ADFI during the finisher period compared to CON pigs, respectively. The marginal differences observed in ADG and ADFI resulted in numerical increases (*P* > 0.10) observed in gain/feed ratio throughout the experiment.

**Table 4.** Effects of a second iron injection administered on days 6 to 8 on pen growth performance^1^

Item	Treatment		SEM	*P*-value
	CON	+Fe		
Average daily gain, kg				
Nursery^2^	0.55	0.56	0.02	0.57
Finisher ^3^^,^^S^	0.91	0.94	0.02	0.29
Wean to end of study^S^	0.81	0.83	0.01	0.32
Average daily feed intake, kg				
Nursery^2^	0.98	0.98	0.03	0.97
Finisher^3^^,^^S^	2.28	2.31	0.05	0.73
Wean to end of study^S^	1.88	1.90	0.04	0.74
Gain/feed				
Nursery^2^	0.56	0.57	0.01	0.11
Finisher^3^	0.40	0.41	0.005	0.41
Wean to end of study	0.43	0.44	0.004	0.33

^1^Data represents 12 pens/treatment with pens stocked at 6 pigs/pen. All pigs received an initial iron injection (200 mg Fe) at birth; the +Fe group received a second iron injection (200 mg Fe) at enrollment.

^2^Nursery period included weaning to week 5.

^3^Finisher period included week 5 to the end of study.

^S^Significant sex effect present where barrows had a greater ADG and ADFI compared to gilts, *P* ≤ 0.05.

Often an improvement in ADG is directly related to an increase in ADFI, mostly due to the increase in the quantity of nutrients ingested likely exceeding the maintenance nutrient requirement and thus permitting more tissue accretion (Whittemore, 1986; Patience et al., 2015). However, in the current study there were only marginal improvements in ADFI for the +Fe pigs. Jolliff and Mahan (2011) reported a greater ADFI from 7 to 21 d-postweaning in pigs injected with an additional iron injection 6 d before weaning (day 17). Furthermore, Estienne et al. (2020) reported that pigs administered an additional iron injection at weaning had an increase in ADFI (days 0 to 49 postweaning) when a higher concentration of copper was supplied in the nursery diet (250 mg Cu/kg diet). In the current study, there were no treatment effects observed for ADFI or gain/feed ratio during the experiment. Despite the lack of statistical evidence supporting differences in ADFI and gain/feed ratio between treatments, the authors speculate that the difference in final BW and the improved ADG for the +Fe pigs must be a function of the accumulation of minor improvements in ADFI and gain/feed ratio over the duration of the experiment. Mathematically, because of the relationship between ADG, ADFI, and gain/feed ratio, a significant change in any of the values must be accompanied by a significant change in another value or smaller, nonsignificant changes in the other two values. Furthermore, because ADFI and gain/feed ratio were analyzed on a pen basis (*n* = 12/treatment) there may not have been enough statistical power to support these minor differences.

### Carcass traits, primal cuts, and pork quality

Carcass response traits of the selected slaughter pigs are provided in Table 5. The +Fe pigs demonstrated an ~2.5% heavier HCW and CCW (*P* = 0.33 and *P* = 0.34, respectively) compared to the pigs from the CON group. The marginal differences observed in HCW and CCW translated to marginal increases in selected back fat depths (first rib, last rib, and 10th rib), carcass length, and an ~2.9 % larger loin muscle area (LMA) for the +Fe pigs (45.32 vs. 44.06 cm^2^, respectively). There were expected sex effects present regarding slaughter weight, HCW, and CCW in which barrows were around 5% heavier compared to gilts at the time of slaughter (116.8 vs. 111.05 kg; *P* = 0.03).

**Table 5.** Effects of a second iron injection administered on days 6 to 8 on carcass traits^1^

Item	Treatment		SEM	*P*-value
	CON	+Fe		
Slaughter weight, kg^S^	113.13	114.72	1.75	0.53
Hot carcass weight, kg^S^	82.75	84.85	1.49	0.33
Cold carcass weight, kg^S^	80.93	82.99	1.48	0.34
Dressing, %	73.15	73.91	0.43	0.22
Shrink loss, %	2.20	2.20	0.06	0.97
Carcass length, cm	81.73	82.37	0.66	0.50
Back fat depth, cm				
First rib	3.37	3.43	0.22	0.84
Last rib	2.16	2.60	0.23	0.19
10th rib^S^	1.82	1.85	0.15	0.88
Last lumbar	1.57	1.50	0.14	0.76
Loin muscle area, cm^2^	44.06	45.32	1.69	0.60
Absolute organ weight, g				
Liver	1,676	1,679	32.4	0.95
Spleen	161	169	7.7	0.47
Relative organ weight, % of body weight				
Liver	1.48	1.47	0.03	0.68
Spleen^s^	0.14	0.15	0.01	0.53

^1^Treatment means are reported as least squares means from 12 pigs per treatment; data is based on the left side of the carcass. All pigs received an initial iron injection (200 mg Fe) at birth; the +Fe group received a second iron injection (200 mg Fe) at enrollment.

^S^Significant sex effect present where barrows had heavier weights compared to gilts, *P* ≤ 0.05.

^s^Significant sex effect present where gilts had a heavier relative spleen weight compared to barrows, *P* < 0.01.

Results for primal cuts are provided in Table 6. There were sex effects present for absolute Boston butt and belly yield where barrows had a ~7% and 9% greater yield compared to gilts. Furthermore, +Fe pigs had a heavier trimmed loin (10.67 vs. 9.95 kg; *P* = 0.04) which was also observed on a relative to slaughter weight basis (9.30 vs. 8.80 %; *P* = 0.03) compared to the CON pigs. The increase in trimmed loin yield is a function of the numerical increases observed for carcass length and LMA.

**Table 6.** Effects of a second iron injection administered on days 6 to 8 on primal cuts^1^

Item	Treatment		SEM	*P*-value
	CON	+Fe		
Absolute primal cut weight, kg				
Boston butt^S^	3.72	3.71	0.08	0.96
Picnic shoulder	3.90	3.98	0.13	0.65
Loin	9.95	10.67	0.23	0.04
Belly^S^	6.34	6.30	0.16	0.87
Spare rib	1.55	1.63	0.04	0.14
Ham	9.04	9.28	0.18	0.35
Relative primal cut weight, % slaughter weight				
Boston butt	3.29	3.23	0.06	0.53
Picnic shoulder	3.44	3.46	0.08	0.85
Loin	8.80	9.30	0.15	0.03
Belly	5.60	5.48	0.10	0.40
Spare rib	1.37	1.42	0.03	0.27
Ham	8.00	8.09	0.12	0.59

^1^Treatment means are reported as least squares means from 12 pigs per treatment; data is based on the left side of the carcass. All pigs received an initial iron injection (200 mg Fe) at birth; the +Fe group received a second iron injection (200 mg Fe) at enrollment.

^S^Significant sex effect present where barrows had heavier primal cuts compared to gilts, *P* ≤ 0.05.

There were no treatment effects present for any pork quality measures (Table 7). There were sex effects present for muscle pH at 24-h and a* (meat color), where gilts had around a 4.8% and 10.7% increase respectively compared to barrows.

**Table 7.** Effects of a second iron injection administered on days 6 to 8 on pork quality^1^

Item	Treatment		SEM	*P*-value
	CON	+Fe		
Muscle pH				
45-min pH	6.09	6.04	0.09	0.73
24-h pH^S^	6.01	6.02	0.06	0.93
Δ pH	0.07	0.02	0.13	0.77
Drip loss, %	9.59	11.15	0.96	0.26
Purge loss, %				
Day 7	3.00	2.96	0.37	0.95
Day 14	5.57	5.48	0.61	0.92
Day 30	8.44	8.79	0.56	0.67
Meat color				
L*	60.12	60.20	1.13	0.96
a*^S^	8.28	8.13	0.27	0.69
b*	18.88	15.56	2.48	0.36
Subjective meat quality^2^				
Color	3.04	3.04	0.17	1.00
Marbling	1.71	1.58	0.20	0.66
Firmness	2.50	2.38	0.24	0.72

^1^Treatment means are reported as least squares means from 12 pigs per treatment; data is based on the left side of the carcass. All pigs received an initial iron injection (200 mg Fe) at birth; the +Fe group received a second iron injection (200 mg Fe) at enrollment.

^2^Color: National Pork Producers Council (NPPC) color scale (1 to 5): 1 = pale pinkish to white; 5 = dark purplish red. Marbling: NPPC marbling scale (1 to 5): percentage fat in the loin muscle. Firmness: NPPC firmness scale (1 to 5): 1 = very soft; 5 = very firm.

^S^Significant sex effect present where gilts had a greater pH compared to barrows, *P* ≤ 0.05.

In summary, administering an additional iron injection before weaning resulted in greater Hb at weaning and the end of the nursery period. The majority of pigs that received only the initial iron injection administered at processing exhibited Hb levels below the critical limit of 11 g/dL and would be considered subclinically iron deficient (Thorn, 2010; Bhattarai and Nielsen, 2015b; Perri et al., 2016). However, pigs receiving the second iron injection on days 6 to 8 demonstrated Hb levels well over the critical limit at weaning demonstrating that an additional iron injection prior to weaning is a viable intervention method that reduces the potential risk of a pig outgrowing their iron stores during lactation and subsequently becoming iron deficient during the weaning and early nursery period. In addition, the additional iron injection resulted in improved growth performance from weaning to the end of the study, which also improved carcass measures associated with the increase in final BW. The work herein suggests that by optimizing Hb at a critical and stressful period such as weaning, may result in a more vigorous pig during the subsequent nursery and grower periods, the sum of which leads to an optimal growth potential permitting improved growth performance and carcass yield. However, further research evaluating the time of intervention (additional injection) relative to the initial injection and weaning is warranted to fully understand the potential benefits of improved growth performance while considering the increase in labor costs associated with providing the additional injection.

## Abbreviations:

ADFI: average daily feed intake
ADG: average daily gain
BW: bodyweight
CCW: cold carcass weight
Hb: hemoglobin
HCW: hot carcass weight
IM: intramuscular
IMPS: Institutional Meat Purchasing Specifications
LMA: *longissimus dorsi* muscle area
NPPC: National Pork Producers Council
NRC: National Research Council
PVC: polyvinyl chloride
SID: standardized ileal digestible
STTD: standardized total tract digestible
USDA: United States Department of Agriculture
